# Six weeks of balance or power training induce no generalizable improvements in balance performance in healthy young adults

**DOI:** 10.1186/s13102-019-0146-4

**Published:** 2019-11-08

**Authors:** Louis-Solal Giboin, Markus Gruber, Andreas Kramer

**Affiliations:** Sensorimotor Performance Lab, Human Performance Research Centre, University of Konstanz, Universitätsstrasse 10, 78464 Konstanz, Germany

**Keywords:** Motor learning, Learning to learn, Strength, Transfer, Sensorimotor, Specificity, Postural control

## Abstract

**Background:**

Training programs for fall prevention often fail to induce large general effects. To improve the efficacy of fall prevention programs, it is crucial to determine which type of training is most effective in inducing generalizable effects, i.e., improvements in untrained situations. Two likely candidates are balance and resistance training. Here, we assessed whether either varied balance training or a training program aiming to increase leg power would improve performance and acquisition rate of a novel balance task.

**Methods:**

Forty-two healthy recreationally active subjects (16 females, age 24 ± 3y) were assigned to a control group, a varied practice balance group or a loaded squat and plyometrics power group, training for 6 weeks (twice per week, 40 min per session). Before and after the training, we measured peak power in countermovement jumps and balance performance in two different untrained balance tasks (10 trials pre and 50 trials post-training).

**Results:**

After training, the performance and the acquisition rate in the two untrained tasks were similar for all groups (no group x time interaction), i.e., no generalization of learning effect was induced by either form of training. Peak power in the countermovement jump did not change significantly in any of the groups.

**Conclusions:**

Neither a six-week power training nor a varied balance training improved performance or acquisition of an untrained balance task. This underpins the task-specificity principle of training and emphasizes the need for studies that assess the mechanisms of transfer and generalization, thus helping to find more effective intervention programs for fall prevention.

## Background

Balance training can induce quick and large improvements in the performance of the balance tasks that were trained. Thus, balance training is widely used for fall prevention [1], or to improve sports performance and reduce sports injuries [2, 3]. However, the effect of balance training on falls in at risk populations remains small. For example, a recent meta-analysis showed limited or even non-significant effects of exercise, including balance training, on falls [4] (but see also [5]). This rather limited impact could be partly due to the fact that, like for most skill training, “learning tends to be quite specific to the trained regimen and does not transfer to even qualitatively similar tasks” [6]. Indeed, in several recent studies and reviews, the quick and large improvement of performance induced by balance training has been shown to be mostly specific to the task trained [7–11], even after months of training [12]. This means that after having trained one balance task (for example, keeping a one-leg stance on one unstable surface such as a slackline), trainees will improve their performance in this task, but will not perform better than control participants in untrained balance tasks (for example, keeping a one-leg stance on a different unstable surface such as a tilt board). This task-specificity effect or lack of generalization might defeat the purpose of balance training for fall prevention or sport performance, since real-life balance challenges may not always be anticipated or trained in the clinic or the gym. Therefore, as stated by Green and Bavelier, one key question in the field of training-induced learning is whether there are training regimens able to induce a generalization of performance improvement beyond the training context, and if yes, by which mechanisms [6]. A transfer effect, i.e. a better performance in the firsts trials, or an increase in the learning rate, i.e. a faster capacity to master a new task, are two possible ways to generalize performance improvements following training. In order to better optimize balance training and to better understand its effect on general balance performance or fall prevalence, more studies specifically dedicated to assess and understand the generalization of balance performance improvement are required.

Growing evidence suggests that balance training doesn’t lead to a faster learning rate of untrained balance tasks [13] and does not lead to transfer of performance [7–11, 14] (but see [15]). However, it must be noted that in the aforementioned studies testing a learning rate effect, the training duration was possibly too short [13], and for the studies testing a transfer effect, the training usually consisted of only one balance task. For visuomotor tasks, it has been shown that training with a broader range of movements may lead to a better generalization and transfer than training with a narrower range of movements [16]. Furthermore, such varied training could also potentially lead to faster learning rate of untrained tasks [17]. Therefore, a varied balance training incorporating many different balance tasks and devices may be the prerequisite for performance generalization to untrained balance tasks. The concept that a varied balance training may induce transfer of performance in untrained balance tasks is in line with previous results [15]. However, in this particular study, the balance training also induced changes in the ankle neuromuscular function that were not significantly different from the changes induced by power training. Therefore, it remains unclear whether the observed transfer of performance was induced by the balance skill training or by the increase in neuromuscular performance. Indeed, there is a large body of evidence supporting a possible link between power and balance performance [18, 19], with some training studies reporting that the increase in neuromuscular performance was associated with improved performance in some of the tested balance tasks [15, 20, 21]. This effect can be explained by the fact that maintaining balance and avoiding falls often requires quick postural adjustments with high rate of force development and high power [18]. In a recent study, we also observed an association between lower limb power and the learning rate of an untrained balance task [13]. However, correlations between power and balance do not necessarily imply a causative link, and multimodal exercise programs are not suitable to elucidate underlying mechanisms.

Therefore, the aim of the present study was to test the generalization effect of two types of training – varied balance training, and leg strength and power training – on balance performance. We hypothesized that both the six-week varied balance training and the six-week strength and power training would lead to a faster acquisition of untrained balance tasks, as well as a transfer of performance. Second, we assessed whether one of the two types of training was superior for performance transfer or increasing the acquisition rate of a novel balance task.

## Methods

### Experimental design

**Fig. 1:**
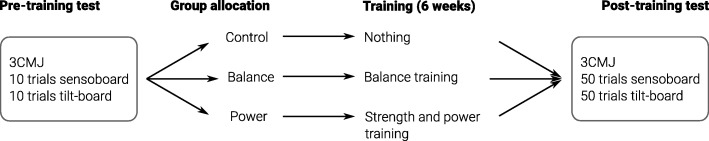
Experimental flow-chart

Performance during two non-trained balance tasks was assessed before and after 6 weeks of training in a control group and two training groups (see also Fig. 1). During the pre-training tests, participants performed 3 maximal countermovement jumps (CMJ) with 1 min of rest in between jumps. Then, they performed 10 trials on each of the 2 tested balance tasks (tilt-board and sensoboard). Afterwards, participants were assigned to one of 3 groups (control, balance or power group), matching group performance for the CMJ and the first trial of the tilt-board and sensoboard task. The rationale behind matching groups according to their pre-training performance instead of allocating them randomly is to reduce pre-training differences between groups, which would render the interpretation of the results much more difficult. At least 24 h after the pre-training test, participants from the power group did a one-repetition maximal strength test in the barbell back squat (1 RM). Then, at least 24 h after the pre-training test or the 1 RM test, participants from the balance and power groups started their balance or power training. The participants from the control group did not train. After 6 weeks of training, participants from the 3 groups did a post-training test. First, participants performed 3 CMJ. Then, participants performed 50 trials on the 2 tested balance tasks (same order as during the pre-training test, order counter-balanced between subjects). The rationale for using 50 trials after the training versus 10 trials before the training was to get a good estimation of the learning curve after the training while limiting the number of trials before the training.

### Participants

Fifty-one young healthy adults (age above 18 years) participated after giving written informed consent. The experiment was in accordance with the regulation of the ethics committee of the University of Konstanz as well as the declaration of Helsinki. Participants were naïve to the tested balance tasks, free from lower limb injuries or balance related impairment. Participants with a national level in a weightlifting or power sport were excluded. Participants were asked to continue their normal sports and physical activity routine during the whole duration of the study (controlled with an activity log). Participants were asked to not participate in any balance training outside from the supervised training. Due to scheduling reasons, 9 subjects dropped out. The final group composition excluding drop-outs can be seen in Table 1. One-way ANOVAs revealed no difference in age (F_2,38_ = 0.25, *p* = 0.78) or weight (F_2,39_ = 2.8, *p* = 0.07). However, a significant difference in height was observed (F_2,39_ = 3.57, *p* = 0.037), and explained by a difference between the balance and the power group (post-hoc Bonferroni corrected *t*-tests, t_39_ = − 2.67, *p* = 0.03).

**Table 1:** Group composition demographics

Group	N	Women	Age (years)	Height (cm)	Weight (kg)
Control	16	7	24 ± 3	174 ± 9	72 ± 12
Balance	14	6	24 ± 3	170 ± 6	65 ± 7
Power	12	3	23 ± 2	179 ± 11	74 ± 13

### Tested balance tasks

**Fig. 2:**
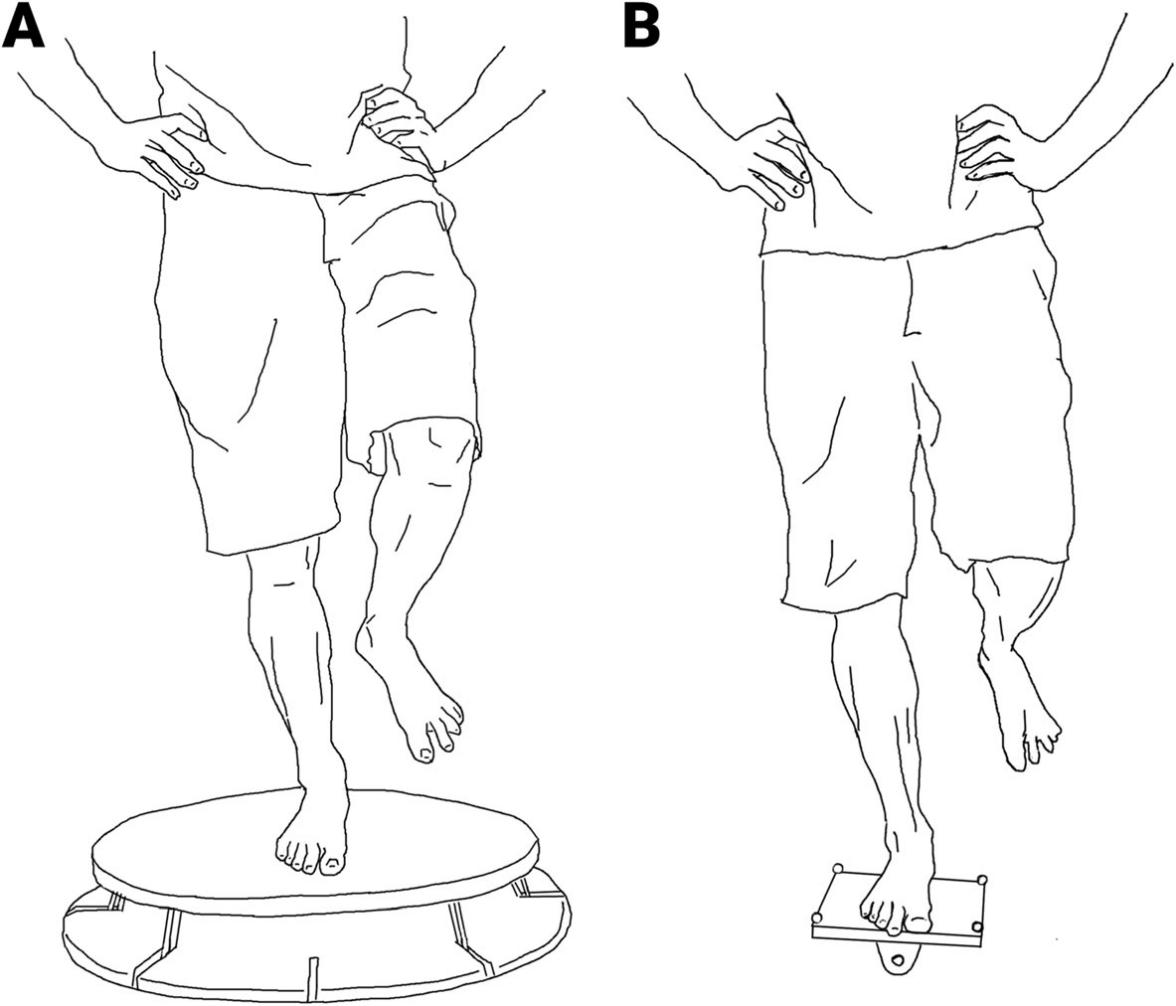
Tested balance tasks. **a** Sensoboard task. **b** Tilt-board task

The two tested balance tasks (tilt-board and sensoboard) were not trained by any of the participants before and during the study. Both tasks were always performed with hands on the hips, and consisted in a one-leg stance with the preferred leg (same leg for both task and pre- and post-training, see Fig. 2). All trials lasted 10 s and were separated by 10 s of rest. There was a break of 1 min 30 s every 10 trials, and a break of 5 min between the 2 tasks. For every trial, an acoustic signal was given 3 s before the start, at the start and at the end. After each trial, a performance feedback estimated with a stopwatch was given to the participant. Performance corresponded to the time at equilibrium during the trial (in s). The tilt-board task consisted of a one-leg stance performed on a custom-made tilt-board with a medio-lateral axis of perturbation. For more details, please see [13]. Briefly, the participant started with a one-leg stance on the tilt-board with one edge of the platform on the floor, and had to bring the platform into a horizontal position for as long as possible during the 10 s of the trial. Performance was measured with motion capture (Vicon Nexus, 12 T40 s camera, 200 Hz), and consisted of the time during which the platform was parallel to the floor (± 5 °). The sensoboard task was performed on a different type of unstable board, with several degrees of freedom (Sensoboard, Sensosports GmbH). Here, the subject started from an elevated platform and stepped with the preferred leg onto the sensoboard platform. The aim was to remain in equilibrium for as long as possible on the board in a one-leg stance (with a performance ceiling of 10 s). The trial started as soon as the non-preferred foot quit the elevated platform. As soon as the board touched the floor or the subject stepped off the board, the trial was terminated. Performance was measured with a stopwatch. We selected these two particular balance tasks assuming that the tilt-board task performance relies more on power (to bring the tilt-board into a horizontal position and maintain it in that position) than the sensoboard task (where the device is already in a horizontal equilibrium position, but is more sensitive to the body sway of the participant).

### Countermovement jump

CMJs were done on a force plate (Leonardo Mechanograph GRFP, Novotec medical GmbH), with hands on the hips. For each jump we calculated the maximal power relative to bodyweight (PmaxRel, W.kg^− 1^). Power was calculated as the product of force and velocity, which was derived from changes in force, with the Leonardo GRFP 4.3 software. For the statistical analyses, the highest value of the three jumps was used.

### Training

Both training types were conducted twice per week, with at least 48 h of rest in between sessions. Prior to each training, participants performed a warm-up consisting of 5 min of cycling on an ergometer with self-selected cadence and power (between 60 and 160 W). The last week of training was a tapering week, in order to limit any interaction between the fatigue accumulation induced by the training and post-training tests. All training sessions were supervised. The training duration of each training session and for each type of training was around 40 min.

### Strength and power training

The strength and power training was adapted from Adams and colleagues, as this program was reported to increase jump height by more than 10 cm [22]. Details of the training can be seen in Table 2. Briefly, the training consisted of barbell back squats and plyometrics. The first day of the week, squats were done prior to plyometrics, and during the second training session of the week, squats were done with lighter weights and after plyometrics. The squat progression followed a classic increase in intensity accompanied by a decrease in volume. The only difference with the training proposed by Adams and colleagues [22], was that we used drop jumps instead of depth jumps and therefore used lower heights than in the original study. The starting height was selected between 20 and 40 cm depending on the participant’s proficiency in the execution of the drop jumps. The height was increased gradually under scrutiny of the trainer (ratio benefits/risks). The assessment of 1 RM was done as follows: Participants warmed up with lower limb stretches and bodyweight leg exercises. Then the coach instructed participants how to perform a barbell back squat. In particular, the thigh had to be parallel to the floor at the low point of the squat. After that, participants started with 1 set of 10 repetitions with only the 20 kg Olympic barbell. Then, under the supervision of the coach, participants increased the barbell weight progressively with 3–5 sets of 1–5 reps. Participants had then 3 trials to reach their maximal weight (5 min rest in between). The average 1 RM value pre-training was 83 ± 31 kg. No injuries were reported during the whole duration of the training. All participants that finalized the 6 weeks of training were able to perform the 2 sets of 2 repetitions at 100% of their initial 1 RM during the penultimate training session.

**Table 2:** Strength and power training

Squat	W1	W2	W3	W4	W5	W6
Day 1	3 × 8 70%	3 × 6 80%	2 × 5 85%	2 × 3 90%	2 × 2 95%	2 × 2 100%
Day 2	2 × 8 50%	2 × 8 60%	2 × 8 70%	1 × 8 70%	1 × 8 70%	Rest
Plyometric	W1	W2	W3	W4	W5	W6
Drop jumps	3 × 10	3 × 10	3 × 8	3 × 8	2 × 6	2 × 4
Both-leg hops	2 × 15 m	3 × 15 m	3 × 15 m	3 × 15 m	2 × 15 m	1 × 15 m
Split-squat walking	2 × 15 m	2 × 15 m	1 × 15 m	1 × 15 m	1 × 15 m	Rest
Split-squat jumping	2 × 10	2 × 8	3 × 6	2 × 6	2 × 6	Rest

Sets × Repetitions. During day 1, squats were done before plyometrics, while during day 2, squats were done after plyometrics. For drop jumps, the goal was to increase height every week. Both-leg hops were done over a distance. Split-squat walking was done over a distance, while split-squat jumping consisted in jumping as high as possible with the fastest transition in leg position possible

### Balance training

The balance training consisted of several commonly used balance tasks and was inspired by the program established by Gruber and colleagues [23]. All balance training trials consisted of 20 s of exercise followed by 40 s of rest. There was always a break of 1 min 30 s between two different balance tasks. Participants trained with 7 different balance devices and therefore trained 7 different tasks. The devices used were: slackline (medio-lateral axis of perturbation, 5 m long, 3 cm wide, Slackline Tools), two different types of BOSU-ball (perturbation in all directions, BOSU balance trainer), Reebok Core Board (its larger axis in the antero-posterior axis of the participant, perturbation in all directions, Reebok), a tilt-board with a semi-hemispheric basis (perturbation in all directions), Posturomed (perturbation in all directions, Haider Bioswing GmBH), and Indo Board (medio-lateral axis of perturbation, Indo Board). All tasks were performed with hands on the hips. For all devices and tasks, the aim was to perform a one-leg or two-leg stance, keeping the device as balanced as possible. The Reebok Core Board, the tilt-board with a semi-hemispheric basis and Indo Board tasks were always started with one side of the platform of the device on the ground, and participants had to bring the platform of the device into the horizontal equilibrium position. The Indo Board task was always performed on 2 legs. In order to increase training enjoyment and motivation, the 7 tasks were alternated: 3 tasks and the slackline task were performed during the weeks 1, 3 and 5, and the other 3 tasks and the slackline task were performed during the weeks 2, 4 and 6. For tasks requiring a one-leg stance, both legs were trained (i.e. 1 training set = 1 set per leg). Performance feedback was given for each trial of the balance training (time at equilibrium estimated with a stopwatch). Details of the training are given in Table 2. The balance training was effective in increasing task-specific performance: for instance, the performance improvement in the slackline task was 260 ± 80% on average for all the participants in the balance training group.

### Analysis and statistics

Statistics were performed with R (R version 3.4.2, the R foundation for statistical computing). We were mostly interested in the influence of group on the speed of acquisition of the tilt-board and sensoboard tasks (i.e. slope of performance across the number of trials performed). Therefore, we tested the interaction between the group variable and the number of trial performed for each task in separate analysis pre- and post-training. For this, we used linear mixed effects models with random intercepts and random slopes for participants and the Satterthwaite’s method to approximate degrees of freedom (lme4 and lmerTest R package). We used fixed effects for the factors group and number of trial, and random effects for subjects. The model also tested the covariance between random intercepts and random slopes by subject. We added random intercepts and slopes by subjects as previous experience on the topic showed us that subjects tend to start at different level of performance and their learning progression can be very variable. Furthermore, this allowed us to maximize the error structure of the model and limit type I errors [24]. However, for the analysis of the post-training data, we could not maximize the error structure of the model by adding random slopes by subject since this addition prevented the models to converge. To test for a potential transfer effect induced by the training, we compared the performance per subject of the 10 trials of the pre-training test with the performance of the first 10 trials of the post-training test between the 3 groups with mixed effects model. We used a model with time and groups as fixed effects (with a time × group interaction) and subjects as random effects (with random intercept and random slope over time by subject). It must be noted that the performance data at pre- and post-training level was not following a normal distribution (as revealed by Q-Q plots). A square root transformation helped the data to reach (for the pre-training performance data on the tilt-board and sensoboard, and for the post-training data on the tilt-board) or get closer to the normal distribution (for the post-training data on the sensoboard). Therefore, the models testing the difference in speed of acquisition between groups and the transfer effect between groups were performed with the square root of the performance. We tested the effect of training on PmaxRel by using mixed effects models analysis to compare PmaxRel pre- and post-training and between groups (with a group × time interaction and random intercept by subject). We used Pearson correlations between PmaxRel pre-training and increase in PmaxRel post-training (PmaxRel post-training in percent of PmaxRel pre-training).

## Results

**Fig. 3:**
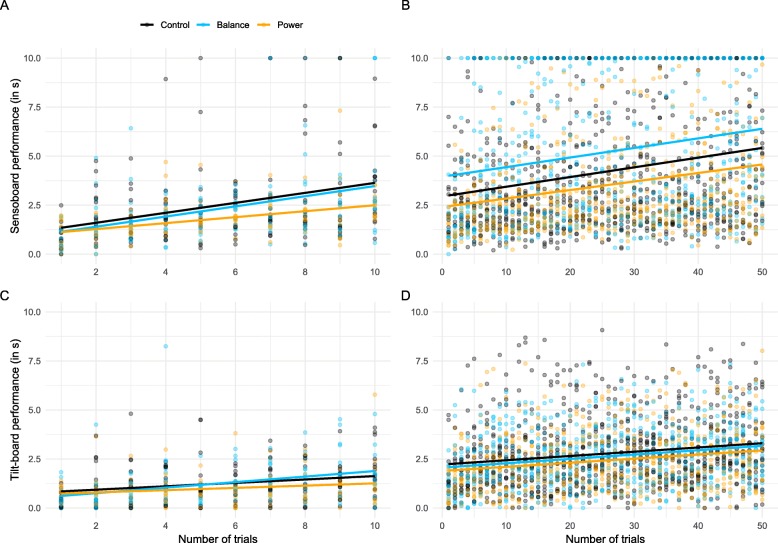
Performance pre- and post-training in the sensoboard and tilt-board tasks. **a** and **c** depict the performance (in s) pre-training for all the trials (10) performed on the sensoboard and the tilt-board respectively for the control (black), balance (blue) and power group (orange). **b** and **d** display the performance (in s) post-training for all the trials (50) performed on the sensoboard and the tilt-board respectively for the 3 groups. A point is fully opaque only when at least 3 points of the same colour are superposed. Solid coloured lines correspond to linear fit for each group

The mixed model analyses revealed only a time effect but no effect of the different trainings for the acquisition of both of the untrained balance tasks (see Table 4 for model estimates and their 95% confidence intervals). For the sensoboard task (Fig. 3b), no effect of group (F_2,58_ = 1.8, *p* = 0.18) or group × trial interaction (F_2,2055_ = 0.59, *p* = 0.94) was apparent, only an effect of trial that demonstrated that all groups significantly improved their performance (F_1,2055_ = 169.8, *p* <  0.001). Similarly, for the tilt-board task (Fig. 3d), we also found an effect of trial (F_1,2054_ = 119.9, *p* <  0.001), but no effect of group (F_2,70_ = 0.35, *p* = 0.70) or group × trial interaction (F_2,2054_ = 0.17, *p* = 0.84).

To make sure that potential differences between groups did not stem from differences that were already present pre training, we also analysed the ten pre training values, with nearly similar results: for the sensoboard task (Fig. 3a), no effect of group (F_2,39_ = 0.25, *p* = 0.78) and no interaction group × trial was observed (F_2,39_ = 0.26, *p* = 0.77), only an effect of trial (F_1,39_ = 46, *p* <  0.001), indicating again a significant increase of performance already during the first ten trials pre training. For the tilt-board task (Fig. 3c), we observed an interaction (F_2,39_ = 3.49, *p* = 0.04) and an effect of trial (F_1,39_ = 43.5, *p* <  0.001), but no effect of group (F_2,39_ = 0.76, p = 0.78). The significant interaction was explained by the lower slope of the power group compared to the balance group (see Table 3).

**Table 3:** Training plan of the balance training

Weeks	W1	W2	W3	W4	W5	W6
Training sets per task	4	4	5	5	5	5
Notes	Use both legs for all tasks during sets 1–3. Use only one leg during set 4.	Same than W1.	Use both legs for all tasks during sets 1–2. Use only one leg during set 3–5. Close eyes during sets 4–5.	Same than W3	All sets are done with a 1 leg stance. Close eyes during set 2. Look upward during set 3. Try to catch a ball sent by the trainer during set 4–5.	Same than W5, but on the second training day of W6, only perform 3 sets per tasks.

**Fig. 4:**
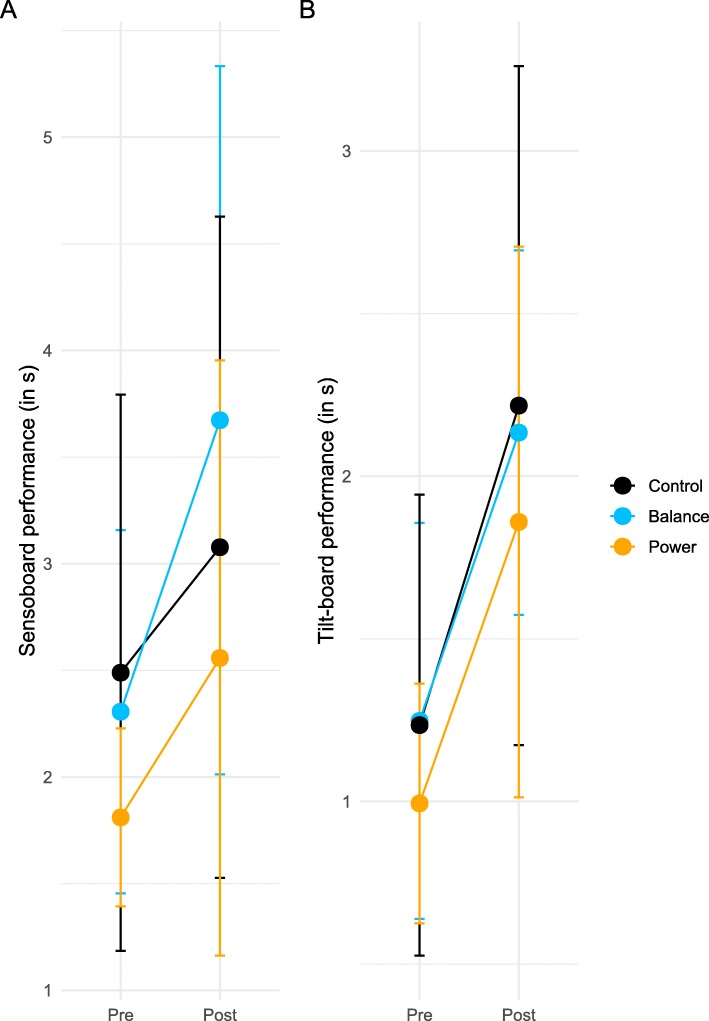
Effect of training on transfer of performance. The averaged performance (in s) of pre-training trials (Pre) and the averaged performance of the first 10 trials post-training (Post) for the control (black), balance (blue) and power group (orange) in the sensoboard (**a**) and tilt-board tasks (**b**). Error bars represent standard deviation

Then, we tested whether a transfer effect on performance was induced by the training, i.e., we compared the performance per subject of the pre-training trials (10 trials) and the performance of the 10 first post-training trials (see Table 4 for model estimates). For the sensoboard task (Fig. 4a), we only found a time effect (F_1,39_ = 27.1, *p* <  0.001) but no group (F_2,39_ = 1.5, *p* = 0.24) or group × time effect (F_2,39_ = 0.97, *p* = 0.39). Similarly, for the tilt-board task (Fig. 4b), we found a time (F_1,39_ = 59.6, p <  0.001), but no group (F_2,39_ = 0.66 *p* = 0.52) or group time × effect (F_2,39_ = 0.04, *p* = 0.96).

**Table 4:** Model Estimates

Data	Fixed effect	Estimate	SE	Lower 95% CI	Upper 95% CI	t-value	*p*-value
sqrt (sensoboard post-training)	Intercept	1.71	0.12	1.47	1.95	13.7	< 0.001***
	Trial	0.0121	0.0015	0.009	0.0152	7.82	< 0.001***
	Group Control	−0.2	0.17	− 0.54	0.12	−1.21	0.23
	Group Power	−0.34	0.18	−0.69	0.01	−1.86	0.07
	Trial: Group Control	−0.00034	0.0021	−0.004	0.004	−0.16	0.87
	Trial: Group Power	−0.00078	0.0023	−0.005	0.004	−0.34	0.73
sqrt (tilt-board post-training)	Intercept	1.26	0.08	1.1	1.41	15.55	< 0.001***
	Trial	0.0081	0.0011	0.006	0.01	6.86	< 0.001***
	Group Control	0.057	0.11	−0.16	0.27	0.51	0.6
	Group Power	−0.038	0.12	−0.27	0.19	−0.32	0.75
	Trial: Group Control	−0.00084	0.0016	−0.004	0.002	−0.52	0.6
	Trial: Group Power	−0.00089	0.0017	−0.004	0.002	−0.5	0.61
sqrt (sensoboard pre-training)	Intercept	1.05	0.089	0.88	1.22	11.77	< 0.001***
	Trial	0.08	0.019	0.04	0.12	4.25	< 0.001***
	Group Control	0.039	0.12	−0.19	0.27	0.32	0.75
	Group Power	−0.05	0.13	−0.31	0.2	−0.39	0.7
	Trial: Group Control	0.00022	0.026	−0.05	0.05	0.008	0.99
	Trial: Group Power	−0.017	0.028	−0.07	0.04	−0.624	0.54
sqrt (tilt-board pre-training)	Intercept	0.64	0.091	0.47	0.82	7.07	< 0.001***
	Trial	0.07	0.012	0.05	0.09	5.84	< 0.001***
	Group Control	0.13	0.12	−0.1	0.38	1.08	0.28
	Group Power	0.14	0.14	−0.12	0.4	1.04	0.3
	Trial: Group Control	−0.027	0.017	−0.06	0.006	−1.58	0.12
	Trial: Group Power	−0.04	0.018	−0.084	−0.01	−2.62	0.012*
sqrt (sensoboard), transfer	Intercept	1.41	0.07	1.28	1.55	20.14	< 0.001***
	Time Post	0.34	0.082	0.18	0.5	4.14	< 0.001***
	Group Control	0.04	0.096	−0.14	0.23	0.42	0.69
	Group Power	−0.13	0.1	−0.33	0.07	−1.25	0.22
	Time Post: Group Control	−0.15	0.11	−0.37	0.07	− 1.33	0.19
	Time Post: Group Power	−0.12	0.12	−0.36	0.11	−1.01	0.32
sqrt (tilt-board), transfer	Intercept	0.97	0.08	0.82	1.13	12.1	< 0.001***
	Time Post	0.38	0.085	0.21	0.54	4.44	< 0.001***
	Group Control	0.013	0.11	−0.2	0.23	0.12	0.9
	Group Power	−0.08	0.12	−0.31	0.15	−0.66	0.51
	Time Post: Group Control	0.022	0.12	−0.2	0.25	0.19	0.85
	Time Post: Group Power	−0.0088	0.12	−0.25	0.23	−0.071	0.94

Fixed effects estimates, standard error (SE), lower and upper 95% confidence interval, t-value and *p*-value. Sqrt corresponds to square root. Intercept corresponds to the intercepts (trial 1) of the reference group (balance group). Intercept is tested against zero. Group Control and Group Power correspond to the difference between Intercept and the intercept of the control and power group. Trial corresponds to the slope of the reference group (i.e. the balance group). Trial: Group Control and Trial: Group Power correspond to the difference between the slope of the balance group (Trial) and the slope of the control and power group

One star indicates a significant difference with *p* < 0.05. Three stars indicate a significant difference with *p* < 0.001

For PmaxRel, we found no time (F_1,39_ = 2.03, *p* = 0.16), group (F_2,39_ = 0.068, *p* = 0.93), or group × time effect (F_2,39_ = 0.13, *p* = 0.87), indicating that there was no significant effect from the different trainings on PmaxRel, and no difference of PmaxRel between groups pre- and post-training (control: 48.37 ± 9.13 W.kg^− 1^ pre-training and 48.93 ± 9.53 W.kg^− 1^ post-training; balance: 49.24 ± 10.65 vs. 49.61 ± 11.14; power: 47.62 ± 7.75 vs. 48.55 ± 7.29). We also tested the correlation between PmaxRel pre-training and the increase of PmaxRel post-training when pooling all subjects together (r = − 0.185, *p* = 0.24), for the strength and power group alone (r = − 0.454, *p* = 0.14), the balance group alone (r = − 0.086, *p* = 0.77), and the control group alone (r = − 0.011, *p* = 0.97).

## Discussion

We did not observe a faster acquisition of the two untrained balance tasks tested after 6 weeks of balance or power training compared to a control group. Moreover, we did not observe an immediate transfer effect on performance compared to a control group, i.e., a better performance in the firsts trials post-training.

The present study expands on previous results where one varied practice session with several balance tasks and devices had no effect on the acquisition or retention of an untrained balance task compared to a control group [13]. Following the theory constructed via experiments with visuomotor tasks [17] or informatics models [25], we suggest that balance tasks are too complex and the possible outcome of each trial too numerous to easily infer the correct motor command of a new task from the past experience constructed with a 6 weeks training of slightly different balance tasks. Therefore, if such a learning to learn effect exists in the context of balance training, a very large amount of past experience (i.e., years) seems necessary to influence the learning efficiency of new balance tasks and induce generalization of effects.

In addition to the lacking effect on the learning rate during the acquisition of a novel task, no direct transfer to the untrained tasks was observed when comparing the first ten trials after the training between groups. This result is in line with previous training studies that were designed to assess the effect of balance training on untrained balance tasks, and found large effects only in the tasks that had been trained [7–10, 12, 14], underpinning the task-specificity principle of balance training. The task-specificity effect observed here can be explained by the task-specific neural adaptations following balance training [26]. The changed neural networks may be so optimised for a particular task that they are not recruited, or have no use for a different task. This absence of transfer following 6 weeks of training can be seen as problematic with respect to the suitability of balance training for fall prevention, as the training has to reduce fall probability in the very first balance perturbation encountered to be functionally relevant. It is possible that the 6 weeks of training used in the present study were too short or did not constitute a high enough training volume to induce generalizable effects. Indeed in a recently updated meta-analysis, Sherrington and colleagues concluded that three or more hours of balance-challenging exercises per week and a total training volume of more than 50 h help to reduce fall rates in some of the elderly populations that were examined [1, 5]. However, the requirement of such a large training dose may constitute a challenge for fall prevention or rehabilitation in clinical settings, where the time available for training tends to be rather scarce, and the delay for beneficial outcomes must be short.

As a side note, even though no transfer effect was seen, there was a large main effect of time, i.e., all groups including the control group improved in the post tests compared to the pre-tests. This effect can be explained, at least partly, by the experimental test-retest paradigm [27]. This effect emphasizes the need for a control group in studies testing the effect of different types of balance training on balance performance [12].

The power training used in the present study did not yield any different results than the balance training, i.e., it did not improve the learning rate in the untrained balance tasks and elicited no transfer effect. However, it must be noted that the learning rate of the power group was lower than the learning rate of the balance group at pre-training level for the tilt board task. Since this flatter learning curve had no influence on the statistical test of transfer effect between groups (see Fig. 4b), we deemed it as a small effect, possibly emerging from the stochastic nature of balance tests, with most probably no large incidence on the other results (i.e. learning curves post-training). In a previous study, we observed a strong correlation between lower limb peak power and the learning rate of a novel balance task [13]. As this correlation might have been a spurious one, we wanted to investigate a potential causal link between power and learning in the present study, using a power training protocol that has been shown to increase lower limb power [22]. We hypothesized that if a causal relationship existed, the power training should also increase the acquisition rate of the novel balance task and induce a transfer. However, the power training used in the present study failed to significantly increase maximal leg power in countermovement jumps, although it increased maximal leg extensor strength. We can interpret these results in several ways. One possible explanation would be that the increase in maximal strength and power of the leg extensors may not have been high enough to elicit effects, either because the training was not long enough or because of a ceiling effect due to the population tested (young sports students with high baseline power). In that case, the effect of power training in healthy older subjects or patients in regard to learning a new balance task might be different and remains to be investigated. Indeed, a strength and power training may have a larger generalization effect on performance on trainees with a power level below a certain functional threshold [18]. Another possible explanation would be that the correlation between peak power during countermovement jumps and balance performance is a spurious one, and that other parameters such as core stability, rate of force development of the muscles encompassing the ankle joint or anatomical proportions inducing advantageous lever arms are better predictors of balance learning and performance. This spurious relationship between power and balance performance could also explain the correlation discrepancies in the literature [20, 28–30].

If neither balance nor power training are efficient ways to facilitate the learning of new balance tasks and induce general adaptations that transfer to untrained tasks, other types of intervention should be tested with respect to the specificity or generalizability of their effects. In addition to the training of the aforementioned qualities (core stability, rate of force development), aerobic training has been suggested to promote neuroplasticity [31], which in turn could facilitate the learning of new balance tasks. In any case, further research efforts are required to better understand the underlying mechanisms of transfer – or lack thereof – following different types of training. This knowledge is crucial for practitioners designing intervention programs that result in generalizable effects in unknown situations with increased fall risk, thus reducing fall rates, and not only improve balance performance in known, trained tasks.

### Limitations

One limitation to take into account when interpreting the present results is the saturation of the performance in the sensoboard task, due to the 10 s time limit per trial (see Fig. 2b). This saturation might mask an effect of the balance training on the sensoboard task acquisition rate, even though it is unlikely to have a strong effect because this ceiling effect was present in all groups. A second limitation is that the power training used in this study was not able to significantly increase peak power in the countermovement jump in the population studied (healthy sports students), despite the observed increased barbell squat strength in all participants. It is possible that the participants’ peak power was already too high at baseline to change after only 6 weeks of training, since the effect of plyometric training on jump height is known to be dependent to the number of training sessions [32]. This hypothesis is underpinned by the correlation result between the pre-training values and improvement post-training of PmaxRel in the strength and power group, which, albeit not significant, shows better improvement for participants with lower baseline power. We suggest that for future balance studies the effect of the training on power production capacity should be measured with more sensitive and specific tests than countermovement jumps. Thus, to elucidate a potential causal relationship between power and acquisition rate, further training studies with clear increases in power would be helpful, preferably also investigating changes in strength, power and rate of force development in movements related to balance performance. Finally, it is important to keep in mind that the present experiment was conducted with young healthy participants. The same training programs might actually yield generalization of training in at risk populations. However, if that is the case, the present results support the idea that this potential generalization effect may not necessarily stem from the skill training per se, but more from its secondary effects such as changes in neuromuscular performance (e.g. power [18]) or changes in psychological performance (e.g. fear of falling [33]).

### Practical implications

We found that neither 6 weeks of balance training with various balance tasks and devices, nor 6 weeks of strength and power training induced an immediate transfer of performance or facilitated the learning of untrained balance tasks in young healthy subjects. The present results add up to the recent body of evidence suggesting that balance is more a sum of task-specific skills than a general capability. This means that when designing a training program that aims to increase balance in a sport-specific or fall prevention context, great care must be taken in the selection of tasks to train. We advise coaches, athletes or medical practitioners to select and train tasks that are as similar as possible to the balance challenges that are likely to be encountered.

## Conclusions

In conclusion, in a young healthy active population, we observed that neither a six-week varied balance training nor a power training led to better immediate transfer to untrained balance tasks or a faster acquisition compared to a control group. This underpins the task-specificity principle of training and emphasizes the need for studies that assess the mechanisms of transfer and generalization, thus helping to find more effective intervention programs for fall prevention.

## Data Availability

The datasets used and analysed during the current study are available from the corresponding author on reasonable request.

## Abbreviations

1RM: One repetition maximal
CMJ: Countermovement jump
PmaxRel: Maximal power relative to bodyweight
